# Erstdiagnose eines Nierenzellkarzinoms über eine Hodenmetastase

**DOI:** 10.1007/s00120-025-02584-8

**Published:** 2025-04-24

**Authors:** Leonhard Buck, Hans Christoph von Knobloch, Jakob Kohler, Fatih Yalcin, Christiane Maria Stuhlmann-Laeisz, Severin Rodler, Jonas Jarczyk, Philipp Nuhn

**Affiliations:** 1 Klinik für Urologie, Universitätsklinikum Schleswig-Holstein – Campus Kiel, Kiel, Deutschland; 2 Institut für Pathologie, Universitätsklinikum Schleswig-Holstein – Campus Kiel, Kiel, Deutschland

**Keywords:** Skrotale Raumforderung, Retrograde Metastasierung, Ablatio testis, Partielle Nephrektomie, Adjuvante Immuntherapie, Scrotal mass, Retrograde metastasis, Orchiectomy, Partial nephrectomy, Adjuvant immunotherapy

## Abstract

Dieser Fallbericht beschreibt den seltenen Fall einer Hodenmetastase als Erstdiagnose bei einem Nierenzellkarzinom. Der Patient stellte sich mit einer progredienten schmerzlosen Schwellung des linken Hodens vor. Nach initialem Verdacht auf einen Hodentumor wurde eine inguinale Ablatio testis durchgeführt, die das Vorliegen eines klarzelligen Karzinoms bestätigte. Weitere Untersuchungen ergaben Hinweise auf ein primäres Karzinom am linken Nierenunterpol, das durch eine partielle Nephrektomie entfernt wurde. Histopathologisch bestätigte sich ein klarzelliges Nierenzellkarzinom. Die Behandlung wurde durch eine adjuvante Immuntherapie mit Pembrolizumab ergänzt. Postoperativ zeigte die bisherige Nachsorge keine Hinweise auf ein Rezidiv.

## Abstract (en)

This case report describes a rare case of testicular metastasis from renal cell carcinoma, which led to first diagnosis of the primary. The patient presented with a progressive painless swelling of the left testicle. After initial suspicion of a testicular tumor, inguinal excision of the testis was performed, which confirmed the presence of a clear cell carcinoma. Further investigations revealed evidence of a primary carcinoma on the left-lower pole of the kidney, which was removed by partial nephrectomy. Histopathologically, clear cell renal cell carcinoma was confirmed. Treatment was supplemented by adjuvant immunotherapy with pembrolizumab. Postoperative follow-up has shown no evidence of recurrence to date.

## Anamnese

Ein 63-jähriger Patient stellte sich über den niedergelassenen Urologen in der urologischen Ambulanz mit hochgradigem Verdacht auf einen Hodentumor vor. Der Patient gab an, über einen Zeitraum von 2 Wochen eine progrediente Schwellung des linken Hodens bemerkt zu haben. Zum Zeitpunkt der Erstvorstellung war der Patient in gutem Allgemeinzustand mit leicht adipösem Ernährungszustand, ohne Vorerkrankungen und ohne Dauermedikation. Der Patient berichtete über keine B‑Symptomatik, eine urologische Vorstellung war bisher nicht erfolgt. Dysurische Beschwerden bestanden nicht, auch eine Makrohämaturie war bisher nicht aufgetreten.

## Klinischer Befund

In der klinischen Untersuchung imponierte eine deutlich tastsuspekte Verhärtung am linken Hoden bei ansonsten unauffälligem Befund. Sonographisch zeigte sich eine deutlich hypervaskularisierte Raumforderung des linken Hodens von ca. 10 × 10 mm Größe bei insgesamt deutlich hypovolämischem Hoden. Laborchemisch ergaben sich keine Auffälligkeiten. Die Hodentumormarker AFP, HCG, β‑HCG und LDH lagen innerhalb des Referenzbereichs.

## Histologie

**Abb. 1:**
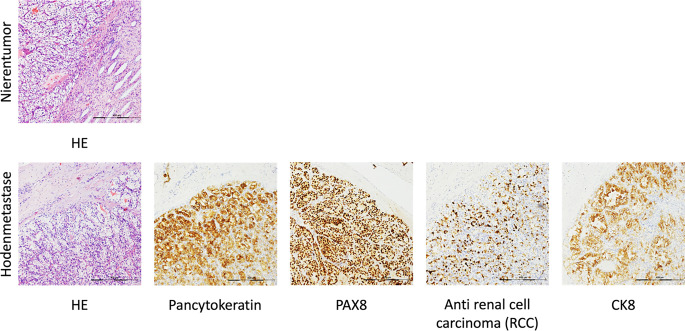
Histologische Bilder des gut differenzierten klarzelligen Nierenzellkarzinoms, Nierenunterpol links (*oben*) und der Metastase im Hoden einschließlich validierender Immunhistochemie (*unten*). Die Bilder wurden mit einem 10 ×-Objektiv aufgenommen. Der Maßstabsbalken *unten rechts* entspricht 400 µm

Nach den üblichen präoperativen Vorbereitungen erfolgte die komplikationslose inguinale Ablatio testis links. Die histopathologische Untersuchung ergab ein klarzelliges Karzinom (WHO-ISUP Grad 2) von 15 mm Durchmesser mit Hämangiosis und Lymphangiosis carcinomatosa in hochgradig atrophem Hodenparenchym. Weiterführende immunhistologische Untersuchungen zeigten, dass die Tumorzellen immunreaktiv für RCC, PAX8, CK8 und Panzytokeratin waren (s. Abb. 1).

## Weiteres Procedere

**Abb. 2:**
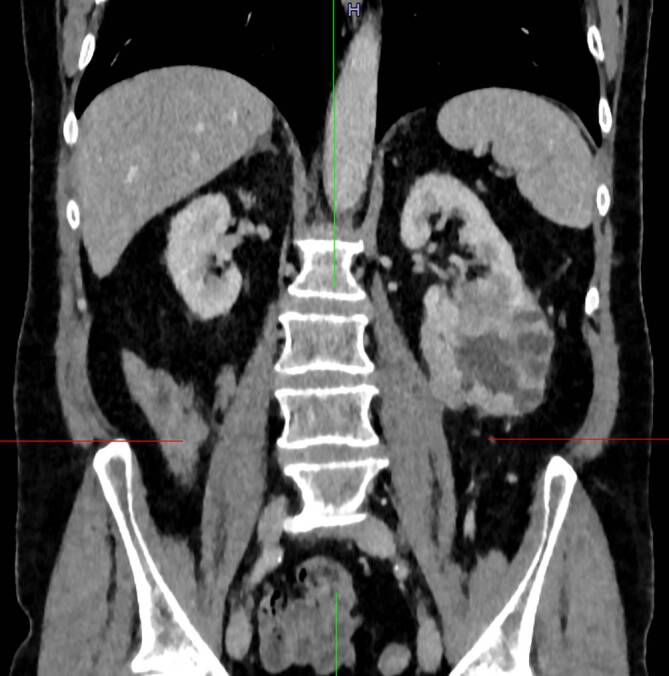
Hochgradig malignomverdächtige Raumforderung am linken Nierenunterpol nach Ablatio testis links

Die postoperativ durchgeführte Staging-Untersuchung mittels CT-Thorax/Abdomen ergab den Verdacht auf eine hoch malignomverdächtige Raumforderung am linken Nierenunterpol (PADUA-Score 8, „intermediate risk“, s. Abb. 2). Weitere Filiae konnten nicht nachgewiesen werden. Nach Vorstellung in unserem interdisziplinären Tumorboard wurde der Patient zur partiellen Nephrektomie links vorbereitet und 2 Monate nach Erstvorstellung erneut operiert. Der endgültige histopathologische Befund zeigte ein Nierenteilresektat des linken Unterpols mit einem maximal 60 mm großen klarzelligen Nierenzellkarzinom (RCC), Tumorformel: pT1b NX L0 V0 Pn0 R0 M1, WHO/ISUP Grad 1 (s. auch Abb. 1). Nach erneuter Vorstellung in unserem interdisziplinären Tumorboard wurde dem Patienten eine adjuvante Immun-Checkpoint-Inhibitor-Therapie mit Pembrolizumab empfohlen. Drei Monate nach partieller Nephrektomie und nach Beginn der adjuvanten Therapie zeigten sich im durchgeführten Staging keine Hinweise auf Lokal- oder Fernmetastasen. Die Therapie mit Pembrolizumab wird aktuell fortgeführt.

## Diskussion

Etwa ein Drittel der RCC wird im metastasierten Stadium diagnostiziert. Typischerweise metastasiert das RCC vorwiegend pulmonal, lymphogen, ossär und hepatisch. Es kann aber auch adrenal, zerebral oder an anderen Orten wie der Bauchspeicheldrüse, dem Rippenfell und der Schilddrüse auftreten [1]. Hierbei sind Hodenmetastasen von RCC extrem selten, in der Literatur sind bis März 2023 nur 51 Fälle beschrieben. In Bezug auf den primären Nierentumor ist wie in diesem Fall der ipsilaterale Hoden am häufigsten betroffen (69,2 %). Eine kontralaterale Metastasierung in den Hoden wurde in 30,8 % der Fälle beobachtet. 3 % der Patienten hatten bilaterale Hodenmetastasen [2]. Die Metastasierung von RCC geschieht vorwiegend hämatogen. Die Ausbreitungswege bei Hodenmetastasen sind nicht eindeutig geklärt. Als Ausbreitungsmechanismus wird eine retrograde venöse Streuung vom primären Nierentumor angenommen [3]. Die Vorstellung der Patienten erfolgt meist mit skrotalen Beschwerden oder einer schmerzlosen Vergrößerung, die Diagnose wird dann durch Ultraschalluntersuchung und eine Orchiektomie bestätigt [4]. Die Hodenmetastasen weisen in der Regel eine klarzellige Histologie auf, die dem primären RCC-Subtyp entspricht. Die Prognose ist unterschiedlich, wobei einige Patienten eine Krankheitsprogression aufweisen, während andere über lange Zeiträume krankheitsfrei bleiben [5].

Mit der Zulassung von Pembrolizumab aufgrund der Daten der KEYNOTE-564-Studie kann Patienten mit testikulären Metastasen eines klarzelligen RCC (ccRCC) nach kompletter Resektion eine adjuvante Immun-Checkpoint-Inhibitor-Therapie angeboten werden [6]. Die Ergebnisse der Studie zeigen einen signifikanten Überlebensvorteil für Pembrolizumab gegenüber Placebo.

Von besonderer Relevanz ist die M1-NED-Subgruppe, d. h. Patienten mit bereits metastasierter Erkrankung, deren Metastasen vollständig reseziert wurden und die nach der Operation tumorfrei waren. Auch in dieser Gruppe zeigte sich ein Hinweis auf einen Überlebensvorteil durch Pembrolizumab, allerdings war die Fallzahl zu klein, um eine statistische Signifikanz zu erreichen. Die aktuelle Indikation für die adjuvante Therapie umfasst Patienten mit hohem Rezidivrisiko, das sich nach vollständiger Resektion von Metastasen ergibt.

Der beschriebene Fall ist der zweite dokumentierte Fall, in dem Pembrolizumab nach Resektion einer Hodenmetastase gegeben wurde, der erste Fall stammt aus dem Interventionsarm der KEYNOTE-564-Studie [7]. Da Hodenmetastasen selten sind, könnte dieser Fall dazu beitragen, das Potenzial der adjuvanten Immuntherapie in dieser spezifischen Patientenpopulation zu verdeutlichen, insbesondere da M1-NED-Patienten möglicherweise am meisten von einer adjuvanten Behandlung mit Pembrolizumab profitieren.

## Fazit für die Praxis

- Hodenmetastasen von Nierenzellkarzinomen sind extrem selten, können aber bei unklarer Hodenschwellung als Differentialdiagnose in Betracht gezogen werden.
- Die Diagnosesicherung erfolgt in der Regel durch bildgebende Verfahren und histopathologische Abklärung nach inguinaler Ablatio testis.
- Entscheidend ist eine interdisziplinäre Therapieplanung (operative Entfernung und ggf. adjuvante Immuntherapie).

## References

[1] Harding G, Cella D, Robinson D, Mahadevia PJ, Clark J, Revicki DA (2007) Symptom burden among patients with Renal Cell Carcinoma (RCC): content for a symptom index. Health Qual Life Outcomes 5(1):34 17570854 10.1186/1477-7525-5-34 PMC1929060

[2] Pliszka A, Rajda S, Wawrzyniak A, Walocha J, Polguj M, Wysiadecki G et al (2023) Testicular metastasis from renal cell carcinoma: a systematic review. J Clin Med 12(17):5636 37685703 10.3390/jcm12175636 PMC10488956

[3] Moriyama S, Takeshita H, Adachi A, Arai Y, Higuchi S, Tokairin T et al (2014) Simultaneous bilateral testicular metastases from renal clear cell carcinoma: a case report and review of the literature. Oncol Lett 7(4):1273–1275 24944706 10.3892/ol.2014.1830 PMC3961255

[4] Dell’Atti L (2016) Unusual ultrasound presentation of testicular metastasis from renal clear cell carcinoma. Rare Tumors 8(3):128–129 10.4081/rt.2016.6471 PMC5064302 27746886

[5] Wang G, Zhou C, Villamil CF, So A, Yuan R, English JC et al (2020) Metastatic renal cell carcinoma to the testis: a clinicopathologic analysis of five cases. Case Rep Pathol 2020:1–6 10.1155/2020/9394680 PMC7073490 32190396

[6] Choueiri TK, Tomczak P, Park SH, Venugopal B, Ferguson T, Symeonides SN et al (2024) Overall survival with adjuvant pembrolizumab in renal-cell carcinoma. N Engl J Med 390(15):1359–1371 38631003 10.1056/NEJMoa2312695

[7] Choueiri TK, Tomczak P, Park SH, Venugopal B, Ferguson T, Chang YH et al (2021) Adjuvant pembrolizumab after nephrectomy in renal-cell carcinoma. N Engl J Med 385(8):683–694 34407342 10.1056/NEJMoa2106391

